# Open Gastrostomy of a Gastric Leiomyoma Proximal to the Gastroesophageal Junction: A Case Report

**DOI:** 10.7759/cureus.59810

**Published:** 2024-05-07

**Authors:** Khristianna M Jones, Jenny B Cherenfant, Gavin H Ward, Andre Nozari, Roynny S Sanchez, Alain Soto, Joshua A Simon, Mohammed M Masri

**Affiliations:** 1 School of Medicine, St. George's University School of Medicine, St. George's, GRD; 2 Department of General Surgery, Delray Medical Center, Delray Beach, USA; 3 Department of General Surgery, Larkin Community Hospital, Miami, USA

**Keywords:** surgical intervention, gastrointestinal bleed, open surgery, laparoscopy, spindle cell tumor, smooth muscle tumors

## Abstract

Gastric leiomyomas are benign, submucosal tumors found incidentally on unrelated imaging or during autopsy. The majority of leiomyomas are asymptomatic; however, patients can develop central ulcerations on the lesions leading to upper gastrointestinal (GI) bleeding. A 75-year-old female, with a past medical history of hypertension, hyperlipidemia, and a cerebrovascular accident, presented with complaints of melena, near-syncope events, lightheadedness, weakness, and hematemesis. A computed tomography (CT) of the abdomen with contrast found a heterogeneous low-attenuation mass of 4×4×3 cm^3^ within the gastric fundus and near the gastroesophageal (GE) junction. After an open gastrostomy and excisional biopsy, the mass was identified as a leiomyoma. This case report reviews the presentation, diagnostic assessments, and treatment of a gastric leiomyoma in a complex location proximal to the gastroesophageal junction. Gastric leiomyomas should be considered as a differential diagnosis for patients presenting with an upper gastrointestinal bleed.

## Introduction

Leiomyomas are benign, smooth muscle tumors from the myometrium and submucosa of monoclonal origin [1]. The most common location for leiomyomas is the uterus. Leiomyomas consist of a pseudocapsule and tumor mass. This mass is made up of areolar tissue and extracellular matrix that includes collagen, elastin, reticular fibers, and spindle cells, which typically exhibit a low mitotic index [2]. Gastric leiomyomas are rare tumors that occur in only 2.5% of all gastric neoplasms [1]; therefore, surgical management of such tumors is sparsely reported in the literature. Ramai et al. (2017) [1] reported a case of an ulcerated gastric leiomyoma in a 68-year-old female patient who was biopsied without excision. We present a case of a 75-year-old female with a gastric leiomyoma located within the gastric fundus and proximal to the gastroesophageal (GE) junction. The tumor was surgically removed via an open gastrostomy with enucleation of the submucosal gastric tumor.

## Case presentation

A 75-year-old female patient, with a past medical history of hypertension, hyperlipidemia, and a cerebrovascular accident, presented with melena, near-syncope events, lightheadedness, weakness, and hematemesis. On physical examination, the patient was in no acute distress. The abdomen was soft, non-tender, and non-distended, and normal bowel sounds were heard on auscultation. Laboratory results revealed a white blood count (WBC) of 8.7 mcL, hemoglobin of 8.7 g/dL, blood urea nitrogen (BUN) of 81 mg/dL, creatinine of 1.2 mg/dL, estimated glomerular filtration rate (eGFR) of 50 mL/minute/1.73 m^2^, lactic acid of 2.2 mmol/L, and positive occult blood in the stool.

**Figure 1:**
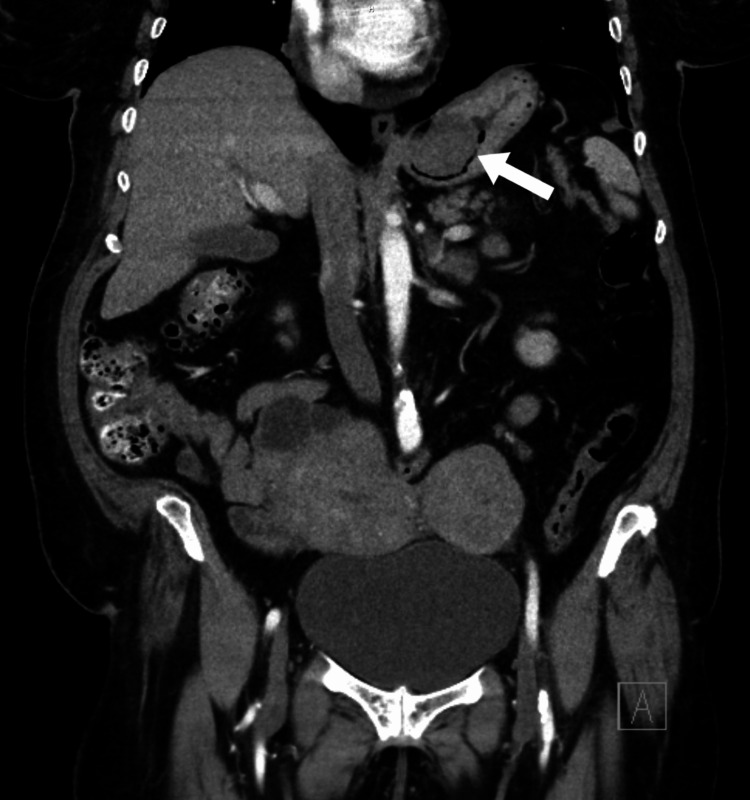
Computed tomography with contrast in coronal plane. Gastric leiomyoma is indicated by the arrow.

**Figure 2:**
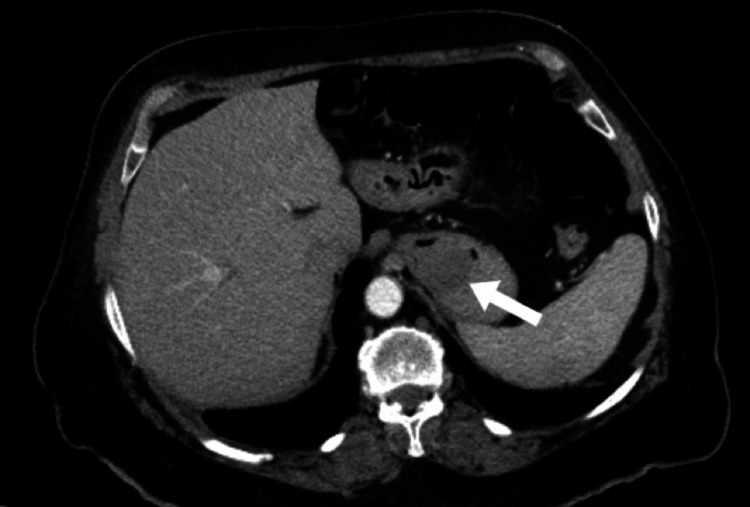
Computed tomography with contrast in axial plane. Gastric leiomyoma is indicated by the arrow.

An esophagogastroduodenoscopy (EGD) demonstrated dried blood throughout the esophagus, stomach, and duodenum. A 3 cm mass covered in fresh blood was identified at the proximal portion of the stomach, within the fundus. The mass was sprayed with hemospray. A biopsy of the mass was not taken at the time due to fear of causing worsening bleeding. Computed tomography (CT) of the abdomen with contrast showed a heterogeneous low-attenuation mass along the gastric fundus measuring 4×4×3 cm^3^ as shown in Figure 1 and Figure 2.

The patient initially underwent surgery for a partial gastrectomy robotically, and the abdominal cavity was explored with no significant alterations noted. The stomach was large, and the area suspected with the tumor was not well-defined from the outside. Manipulation of the stomach did not provide insightful information on the location of the tumor. Then, a decision was made to perform an EGD transoperatively. Retroflexion maneuvers were performed, and the tumor was visualized close to the GE junction. The lesion appeared submucosal and with an ulceration at the distal portion. The position of the lesion that was close to the GE junction was considered too risky to perform the partial gastrectomy robotically. This led to the decision, perioperatively, to abort the robotic approach of the case and to convert it to an open gastrostomy with enucleation of the submucosal gastric tumor.

Immediately after opening the anterior wall of the stomach, the tumor was identified and was covered by mucosa. Enucleation maneuvers were performed, dissecting the tumor from the stomach mucosa. The consistency of the tumor was hard, mostly avascular, with regular edges, and no neoformation of vessels was noted. The tumor was resected satisfactorily. The pathology of the submucosal mass revealed bundles of smooth muscle spindle cells. Immunohistochemical analysis was positive for desmin, muscle actin, beta-catenin, and vimentin and negative for CD1 (c-kit), DOG1, S100, CD117, and Ki-67. These findings were consistent with leiomyoma. The patient tolerated the procedure well, and there were no complications. A wound vacuum was placed over the wound.

Six days after the procedure, the patient experienced severe watery diarrhea for two days, which improved with loperamide. After seven days, the wound vacuum was removed, and an abdominal binder was applied for additional abdominal support. The patient was discharged from the hospital and to follow-up in two weeks as an outpatient. Due to the benign nature of gastric leiomyomas, it was not clinically necessary for the patient to follow up with oncology. The patient was instructed to follow up as an outpatient for continued monitoring with gastroenterology and wound care management.

## Discussion

Gastric leiomyomas are rare tumors representing approximately 2.5% of all gastric tumors [1,3]. This mass is often encountered in individuals aged 50-70 years old, with no gender predilection [1]. They are commonly found in the cardiac region of the stomach; however, they have also been found in the antrum and fundus [1,4]. The mass is slow-growing and can vary in size between 1.3 and 4.7 cm in diameter. The European Society for Medical Oncology and the Japan Gastroenterological Endoscopy Society (JGES) suggest that patients with submucosal tumors < 3 cm should have a follow-up EGD or endoscopic ultrasound within 1-2 years after initial examination [5]. However, lesions greater than 3 cm typically present with central ulceration that can lead to upper gastrointestinal bleeding, dyspepsia, and epigastric pain [6]. This presentation can prompt an EGD that will show a smooth, well-defined mass with mucosal folds overlying the lesion, which is known as Schindler's sign [1].

Gastric leiomyomas are often discovered incidentally during an unrelated abdominal CT scan or post-mortem during an autopsy [7]. Interventions for gastric leiomyomas arise when the patient becomes symptomatic [3]. This patient in the case report presented with an upper GI bleed associated with near syncope, lightheadedness, and weakness. Although other complications such as torsion of pedunculated tumors, intra-peritoneal discharge, hemorrhage, and gastric volvulus may occur, they did not appear in this patient [3,8]. The standard surgical treatment for gastric leiomyomas is wedge resection either by open surgery or laparoscopically [1,6]. The location and size of the leiomyoma influence which approach will be used. Naz et al. (2019) [3] discuss the current laparoscopic methods for resection, which include laparoscopic intragastric resection for tumors not suitable for wedge resection, laparoscopic wedge resection of the gastric wall with endo-stapling guns, and laparoscopic gastrostomy and resection. Laparoscopic wedge resections have become more advantageous for the patient by decreasing postoperative complications such as atelectasis and pneumonia [3,4,9]. In a report by Llorente (1993) [10], it is also argued that the standard surgical treatment of leiomyomas is local excision with a 2-3 cm margin of the surrounding gastric wall. The patient described in this report was initially planned to undergo a robotic partial gastrectomy, but after intraoperative findings, the tumor was in a complex position. To avoid damage to the gastroesophageal (GE) junction, the operation was converted to an open gastrostomy with enucleation.

On immunohistochemical analysis, the tumor was positive for desmin, muscle actin, beta-catenin, and vimentin and negative for CD1 (c-kit), DOG1, S100, CD117, and Ki-67. The pathology of the submucosal mass revealed bundles of smooth muscle spindle cells. These findings are consistent for leiomyoma [1,4]. Immunohistochemical markers help differentiate a leiomyoma from a gastrointestinal stromal tumor (GIST), which may display malignant potential [1]. Unlike leiomyomas, GISTs are positive for CD34 and CD117. Leiomyosarcomas are far less common and can be differentiated from leiomyomas by their high mitotic rate [1]. Growth factors, such as vascular endothelial growth factor (VEGF), are also involved in the pathogenesis of leiomyomas, which is necessary for tumor growth [11]. Because leiomyomas have no malignant potential, the tumor microenvironment is usually better, which leads to a better prognosis [12].

Patients taking nonsteroidal anti-inflammatory drugs (NSAIDs), corticosteroids, and anticoagulants are at an increased risk for GI bleeding [1]. In this case, the patient chronically used aspirin to reduce the recurrence of a cerebrovascular accident. Other complications are often caused by local tumor invasion, bulk symptoms of the tumor (volvulus, torsion, and hemorrhage), or perforation (intra-peritoneal discharge) [3]. These varied presentations suggest that gastric leiomyomas should be considered as a differential diagnosis when signs of upper gastric bleeding are identified [7].

## Conclusions

Gastric leiomyomas are rare, benign tumors that are typically asymptomatic and found incidentally on imaging. Gastric leiomyomas are slow-growing and are usually asymptomatic. However, depending on the size and vascular growth, patients may present with abdominal pain and hematemesis as identified in this case report. This case report also highlights the complexity of a gastric leiomyoma found close to the GE junction. Although leiomyomas do not usually present an acute danger to patients, the complex location of this patient's tumor posed an immediate threat for bleeding, obstruction, or chronic malnutrition that requires surgical intervention. Complex presentations like this case should be considered in the management and treatment and should also be considered as a differential diagnosis of upper gastrointestinal bleeding.
